# Reply of Dr. Faught

**Published:** 1897-10

**Authors:** L. Ashley Faught

**Affiliations:** Philadelphia, Pa.


### Domestic Correspondence.

### REPLY OF DR. FAUGHT.

PHILADELPHIA, PA., September 10, 1897.

TO THE EDITOR:

DEAR SIR,—As my honor has been assailed by the letter published on page 540 of the August number of your journal, its protection now requires that I should state to the profession that every word and statement contained in my letter published in the July issue, page 482, is according to the stenographer's notes of the meeting in question, a copy of which I have in my possession, certified to by the signature of the official stenographer. I wish also to say that all the inferences to be drawn from any other publication that I at any time attempted to falsify the records of the Society are not founded on facts.

Sincerely yours,

L. ASHLEY FAUGHT.

[It has been with some regret that we have felt obliged to admit this personal controversy to our pages, and now, having given both sides ample hearing, the matter must close so far as this journal is concerned.—ED.]

